# Blind source separation analysis of PET dynamic data: a simple method with exciting MR-PET applications

**DOI:** 10.1186/2197-7364-1-S1-A28

**Published:** 2014-07-29

**Authors:** Ana-Maria Oros-Peusquens, Nuno da Silva, Carolin Weiss, Gabrielle Stoffels, Hans Herzog, Karl J Langen, N Jon Shah

**Affiliations:** Institute of Neuroscience and Medicine, Forschungszentrum Jülich GmbH, 52425 Jülich, Germany; Department of Neurosurgery, University Hospital Cologne, 50924 Cologne, Germany; Jülich-Aachen Research Alliance (JARA) – Section JARA-Brain RWTH Aachen University, 52074 Aachen, Germany

Denoising of dynamic PET data improves parameter imaging by PET and is gaining momentum. This contribution describes an analysis of dynamic PET data by blind source separation methods and comparison of the results with MR-based brain properties.

Five glioblastoma patients underwent a 1-hr simultaneous MRI and dynamic FET PET measurement as part of preoperative evaluation and planning of brain surgery.

The MRI protocol included T1-weighted scans acquired before and after contrast agent administration, highlighting the vasculature network and blood-brain barrier breakdown, as well as a T2-weighted scan for oedema visualization and segmentation.

The PET data were binned into 18 consecutive time points and time activity curves of FET uptake were generated.

For the analysis of dynamic PET data, we assumed there are a set of independent sources in the data and used the algorithm svd implemented in Matlab for decomposition. The time activity curves for each source/component were investigated; images representing the components were obtained. The analysis was performed separately on tissue affected by the tumour and on ‘normal tissue’ (complementary of tumour region).

Results obtained on the whole brain and on ‘normal tissue’ were very similar and very similar between patients. The TACs obtained from tissue affected by the tumour (mask defined from SPACE data) were different from those of normal tissue and showed greater variability between patients.

The TAC of component(2) was found to be suggestive of vasculature behavior. This was confirmed by the spatial correspondence between component(2) and the vascular network identified by MRI (from pre- and post-contrast MPRAGE). The effect of denoising on PET data and on the TACs was found to be substantial.

By this method, brain networks can be investigated in (denoised) dynamic data obtained with any PET tracer. For their further identification, comparison with high-resolution MRI data is crucial.

